# Comprehensive Review and Case Study on the Management of Buried Penis Syndrome and Related Panniculectomy

**Published:** 2018-02-01

**Authors:** Hadley Burns, J. Stephen Gunn, Saeed Chowdhry, Thomas Lee, Steven Schulz, Bradon J. Wilhelmi

**Affiliations:** Division of Plastic and Reconstructive Surgery, Hiram C. Polk Jr. M.D. Department of Surgery, University of Louisville School of Medicine, Louisville, Ky

**Keywords:** buried penis, panniculectomy, reconstruction, penis externalization, penoplasty

## Abstract

**Objective:** This paper discusses the various surgical techniques and outcomes associated with management of buried penis syndrome. **Methods:** Presented is the case of a 49-year-old man with morbid obesity, leading to massive panniculus and buried penis. We review our technique for reconstruction of the buried penis and treatment of the overlying large panniculus. Literature search was conducted to review current techniques in correcting buried penis syndrome. **Results:** The patient underwent a successful panniculectomy with removal of all excess skin and tissue. Thoughtful planning and coordination between plastic surgery and urology were paramount to externalize the penis for an excellent functional and cosmetic result. **Conclusions:** Management of a buried, hidden penis is complex and difficult. Patients are often obese and have poor hygiene due to the inability to cleanse areas that are entrapped by excessive fat. Following removal of the overhanging panniculus, satisfactory reconstruction of a hidden penis is possible when proper care is taken to adhere the base of the penis to the pubis. Split-thickness skin grafts are often necessary but depend on the viability of the penile skin and whether it is restricting penile length. Complications with wound dehiscence and infection are not uncommon; however, patients generally recover well, are satisfied with results, and are reported to have fully regained urinary and sexual functions following surgical correction of the buried penis.

Unlike the congenital buried penis seen in pediatric patients, the adult buried penis is an acquired condition highly associated with obesity and infection.^1^^,^^2^ Patients present with an overhanging suprapubic fat pad that either partially or totally conceals the glans penis.^3^^,^^4^ Common complaints include difficulty voiding with a direct urine stream, inability to void standing, complete loss of sexual function, and inability to maintain proper hygienic care of the area.^1^^,^^3^^-^5

Urine discharge from the buried urethral meatus drips over the scrotum and the thigh,^1^ leading to excessive moisture and bacterial overgrowth. This can cause diseased shaft skin and scar contracture of the penile skin to the point where the penis cannot be manually retracted.^3^^,^^5^^-^7 In addition to the physical presentations associated with a buried penis, patients often report experiencing depression and suicidal thoughts.

When massive scrotal lymphedema infection is the cause of buried penis, it is most likely secondary to infection that causes obstruction or aplasia of the draining lymphatic system. This massive lymphedema encases the penis. The most common infections are lymphogranuloma venereum and filarial infestation with *Wuchereria bancrofti*, which are both more commonly seen in African and Asian populations and rarely in the Western world.^2^

It is vital that reconstructive surgery be performed on these patients, not only to relieve them of their physical and mental debilitating symptoms but also because this condition will not correct itself as it often does in children.^4^ Even with substantial weight loss or weight loss surgery, the overhanging suprapubic pad that is secondary to obesity remains in adults,^3^^,^^7^ and there have been no reports of nonsurgical corrections of this condition. When active infection is the cause, medical and surgical routes must be taken. This article discusses the various surgical techniques and outcomes associated with management of buried penis syndrome.

## PATIENT DATA

**Figure 1:**
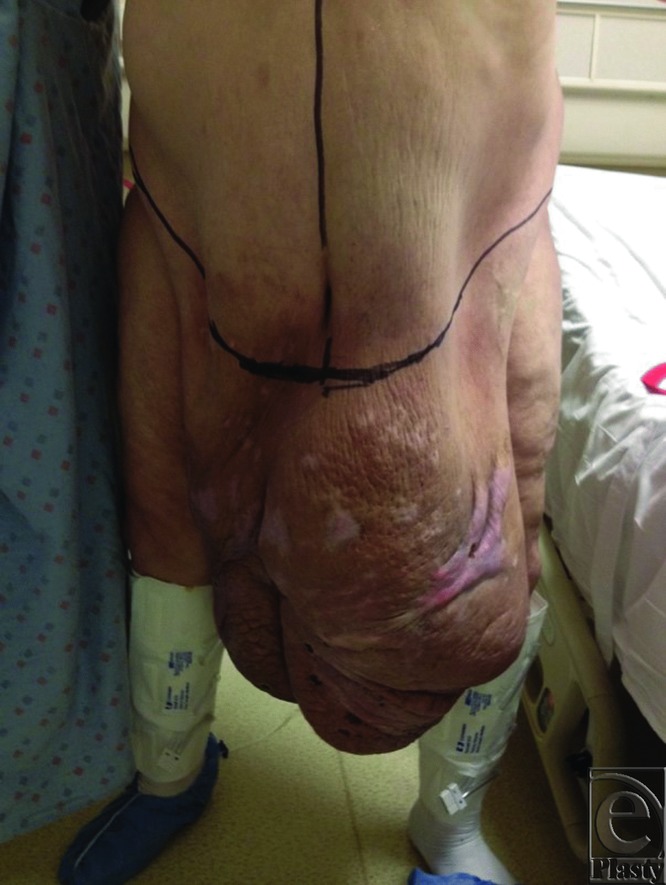
Panniculus encompassing the penis and the scrotum and extending below the knees (Anterior).

**Figure 2:**
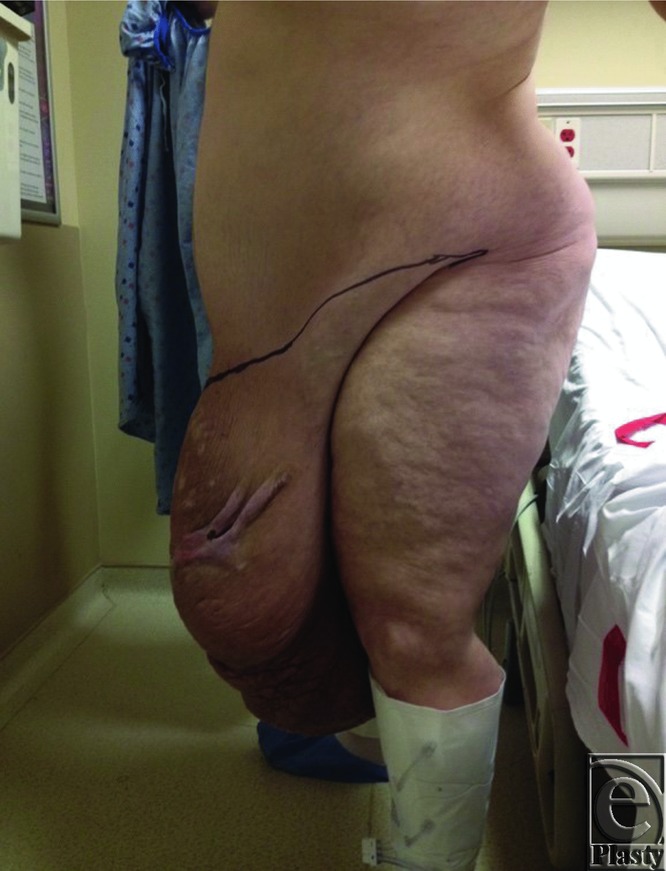
Panniculus encompassing the penis and the scrotum and extending below the knees (Lateral).

**Figure 3:**
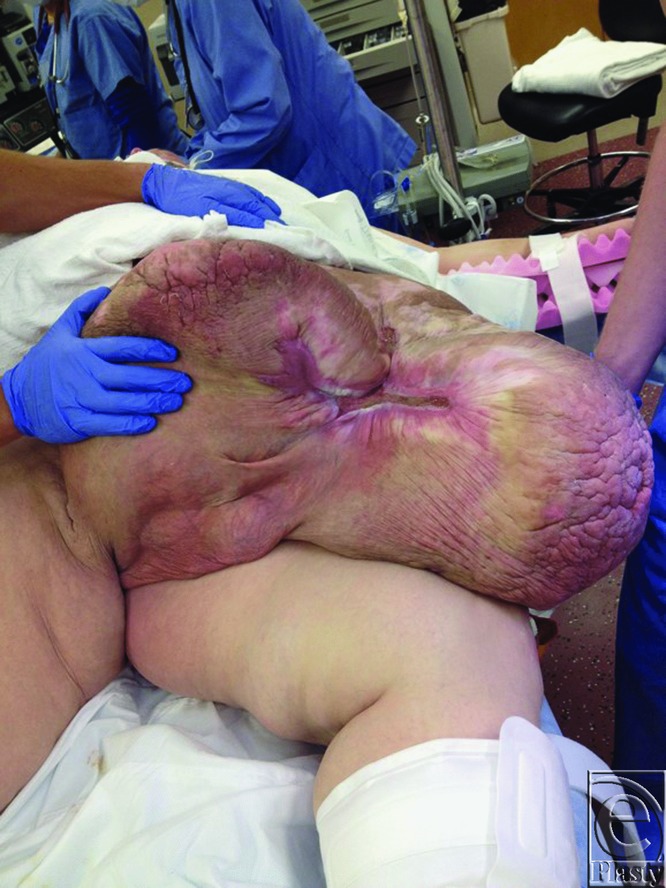
Largest wound on patient, 15 cm in length and 3 cm in width.

**Figure 4:**
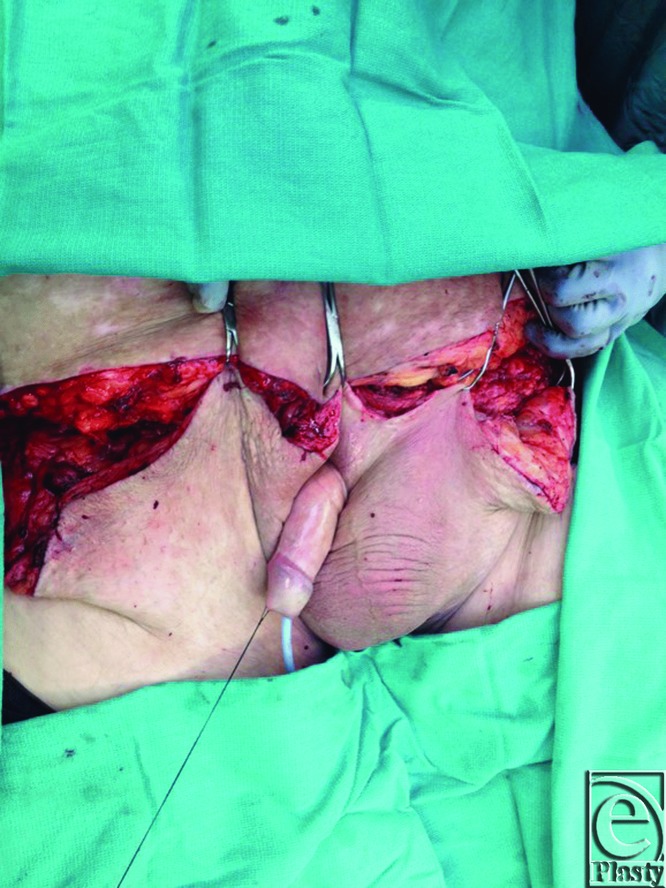
Status post removal of excess skin with resulting penis externalization.

**Figure 5:**
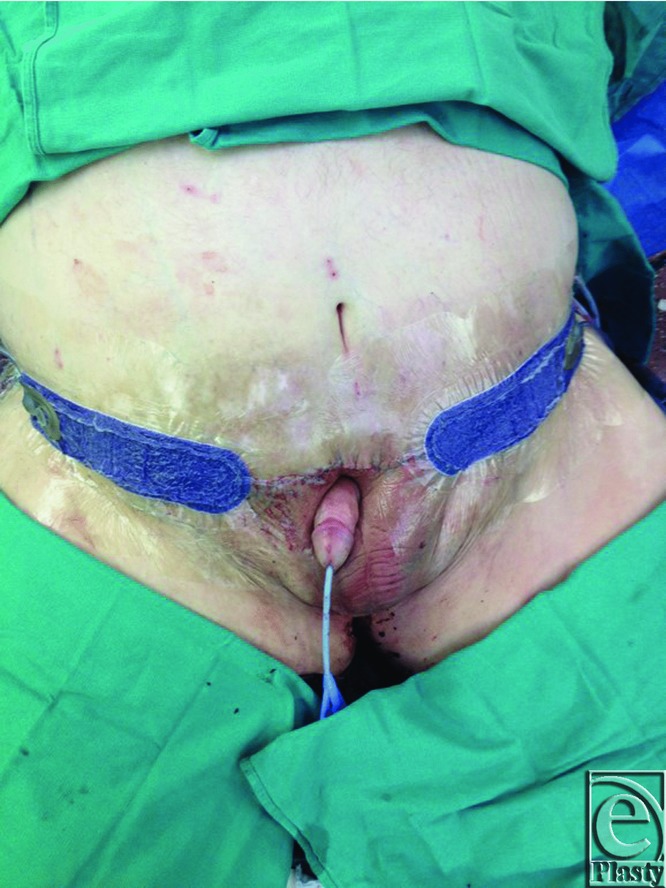
Status post final reconstruction.

Patient S.J. was a 49-year-old man with a history of hypertension and morbid obesity. He suffered from multiple attempts to lose weight in the past but had continued to wax and wane in his weight loss. At the time of examination, he was 5 ft 10 in tall and weighed 400 lb corresponding to a body mass index (BMI) of 57.4. He had recently lost approximately 100 lb, and his panniculus began to descend further, incorporating his penis and scrotum, and extending to below his knees (Figs 1 and 2). Infections and chronic wounds had become more problematic and at the time of examination, he had multiple open wounds, with the largest being approximately 15 cm in length and 3 cm in width (Fig 3). Although his penis was buried and had multiple infections, he was able to void independently using special techniques to empty his bladder completely. A panniculectomy was planned in conjunction with urology performing a penoplasty. The patient underwent a successful panniculectomy with removal of all excess skin and tissue. The penis was externalized for an excellent functional and cosmetic result (Figs 4 and 5). Thoughtful planning and coordination between plastic surgery and urology are paramount to achieve a successful result.

## DISCUSSION

**Figure 6:**
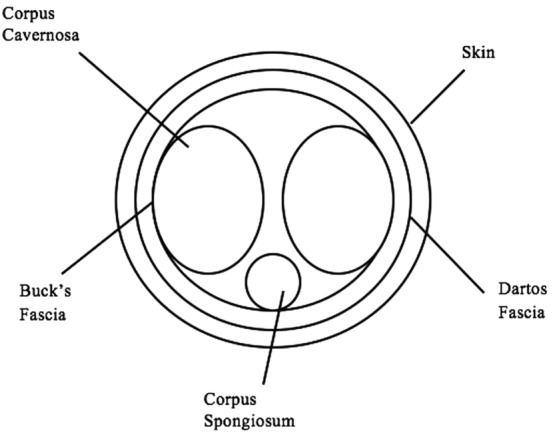
Model of penile anatomy.

**Figure 7:**
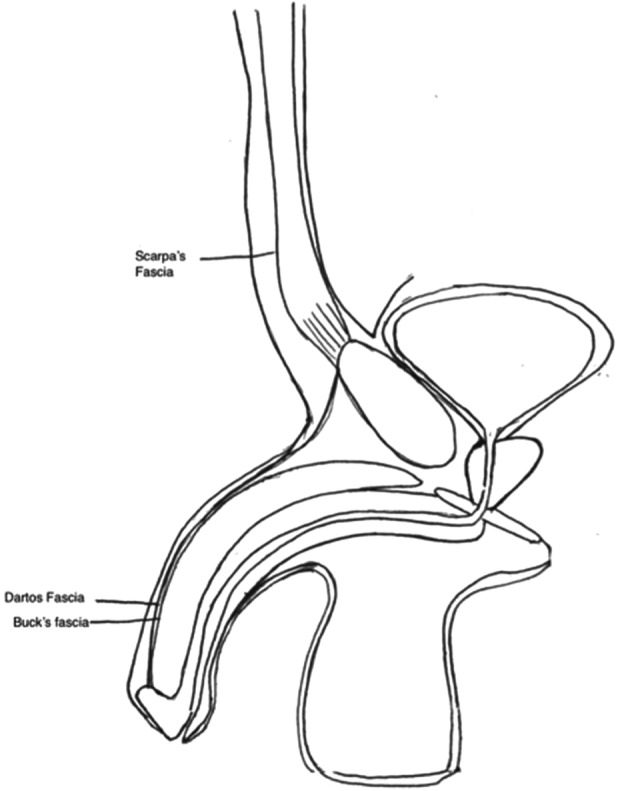
Sagittal model of fascia relationship.

Thorough anatomy of the penis is needed to fully understand the pathology inherent to buried penis syndrome. Buck's fascia immediately encompasses the corpus cavernosum and corpus spongiosum, with Dartos fascia being superficial to Buck's fascia and is continuous with Scarpa's fascia of the abdomen (Figs 6 and 7). There are 2 places of condensation of the overlying penile tissue that protect the penis from retracting or being “buried” by the overdraping skin. That is the condensation of Buck's and Dartos fasciae to pubic symphysis and Scarpa's fascia tethering to the anterior rectus fascia. The following case reviews of buried penises utilize various definitions of what is considered to be overhanging skin or fat pads, and those definitions are addressed when logical.

In general, buried penis syndrome has been defined as a penile shaft buried below the surface of the prepubic skin and can be partially or totally obscured secondary to obesity or lymphedema. While the adult buried penis is primarily due to obesity, those presenting without obesity often present with complications including radical circumcision, severe scrotal lymphedema, penile glans shaft adhesion due to lichen sclerosus et atrophicus, or unusual thickening secondary to peripenile inflammation.^1^^,^^4^

When massive scrotal lymphedema is present, the management should be guided by cause. First, a complete history should be obtained. Patients should be questioned on recent travel to Asia or Africa, which are areas endemic to lymphogranuloma venereum and *Wuchereria bancrofti* infestation, the most common infections seen in massive scrotal lymphedema. A history of recurrent epididymitis has also been seen to play a role. Antibiotics are adequate in acute infections and can reverse the lymphadenitis. When infection is not the cause, other causes include fluid overload secondary to congestive heart failure or sarcoidosis, in which diuretics and steroids are appropriate, respectively. If the lymphedema is chronic and the skin and subcutaneous tissue is fibrotic, surgery must be considered.^2^

With buried penis syndrome, the concealed or covered penile skin is at risk for inflammation, leading to contraction and entrapment of the penis as well as meatal stenosis.^3^ Patients with a buried penis generally present classically in that they are unable to have intercourse and void out of a dimpled hole over their buried penis. They have to sit to micturate and have poor hygienic care due to the inability to reach the entrapped penis and underlying fat folds. The condition is extremely impairing, both physically and mentally, as most patients report depression and suicidal thoughts; thus, urgent management is imperative.^1^^,^^8^

Since many patients undergoing surgery for a buried, hidden penis are obese or super obese (BMI >30 and BMI > 50), it must be noted that studies have shown that they would rather risk the possibility of death in surgery than live with an overhanging panniculus^9^ or entrapped penis. This is a testament to the fact that the quality of life is so deteriorated with this syndrome. Decongestive therapy before resection of a panniculus has been noted to decrease complication rates. Decreased blood transfusions and wound complications have been seen with therapy including manual lymph drainage, lymphatic exercise, education in lymphedema self-management, and compression management.^10^

There are various techniques used in the management of a buried penis and multiple risk factors to consider. Weight loss management has been approached in many cases, but it is not a permanent or satisfactory fix because the mons panniculus often does not dissipate with weight loss.^3^ Surgical management is the only permanent solution in obtaining a functional penis and aims to remove the overdraping mons panniculus or abdominal skin and oftentimes diseased skin surrounding the penis. Surgery is also the only choice if there is massive scrotal lymphedema secondary to constriction of the lymphatic system, usually from infection. In either case, if penile skin is diseased and removed, a split-thickness skin graft is then transferred to the newly visible glans penis. It must also be noted that with surgical correction, Rybak et al^8^ reported a statistically significant increase in quality of life postoperatively (*P* = .021) and a decrease in self-reported postoperative clinical depression (*P* = .035). Surgical correction is thus necessary for not only anatomical correction of buried, hidden penis but also mental improvement.

Since weight loss attempts are unsuccessful at removing the overhanging panniculus, many of the patients undergoing such surgical procedures are still considered obese (BMI > 30), and it must not be forgotten that the more obese the patient, the higher the risk for complications. Whether or not the overhanging panniculus will be removed with weight loss, it is still imperative to educate and advise these patients on increasing physical activity, improving diet, and achieving weight loss before surgery to minimize obesity-related surgical complications. Such complications include wound dehiscence, deep vein thrombosis, and lymphatic dependency.^1^^,^^8^^,^^9^

Risk factors specifically relating to the correction of a buried penis include a predisposition to erectile dysfunction^5^ and loss of sensation over the skin-grafted area for those presenting with eroded penile shaft skin secondary to inflammation and bacterial infections.^3^^,^^5^^-^7 It is possible that sensation to the skin grafted area may return with time, but no timeline has been determined or reported.^4^ To prevent future retraction of the penile skin into the pubic space, it is crucial that fixation of the pubic skin at the penopubic junction and the ventral skin at the penoscrotal junction occurs in order to stabilize the corporeal bodies and skin as a unit.^3^^,^^8^ The following paragraphs go into specific attempts at correcting buried, hidden penises.

In the management of 5 patients with buried penis syndrome, Tang at el^1^ reported 5 successful results, specifically “excellent cosmetic results, with successful and lasting unburying achieved.” The overall procedure included unburying of the glans penis with a circumcising incision, scrotoplasty, combined escutcheonectomy, defatting of the suprapubic pad, and split-thickness skin graft coverage for the defected penile skin. A circumcising incision was performed because the penile skin was scarred and needed to be excised down to the level of Buck's fascia. If the glans has satisfactory shaft skin, and limited shortening, it is possible to deliver the glans by making a ventral slit through the phimotic surface tissue.^8^ Complications were reported with wound dehiscence in the suprapubic wounds of 2 patients, and 1 patient had inflammation and swelling after covering the eroded penile skin with healthy native penile skin. This was eventually fixed in a second surgery using a split-thickness skin graft. Sexual function was possible in all patients, but the time until sexual function returned was not directly measured.^1^

In the case of 37-year-old patient with obesity-related buried penis syndrome secondary to Prader-Willi syndrome, management was similar to that described in the study by Tang et al.^1^ This patient presented classically with a large abdominal panniculus and mons panniculus, as well as meatal stenosis and inability to retract the penis due to contraction of the corona. Management included a limited panniculectomy, excision of diseased penile skin, penile fixation to the pubic symphysis, scrotoplasty, and split-thickness skin graft to the penile shaft. The panniculectomy incision, however, was a modified trapezoid, as opposed to a transverse curved incision. The incision at the inferior midline was created 2-cm cephalad to the junction of the penis so that the skin at the base of the penis could be adhered to the pubic symphysis.

Rybak et al^8^ created a fusiform incision during the management of 11 patients. The incision is taken 12 to 15 cm transversely and 6 to 8 cm vertically at the midpoint.^8^ Excision is carried down to the rectus abdominus fascia, avoiding the spermatic cord and the upper waistline sulcus.^1^^,^^3^^,^^8^ If the waistline sulcus is incised, the abdominal panniculus could potentially encapsulate the penis; thus, caution must be taken to avoid this.^3^

Following the removal of the overhanging panniculus and exposure of the penile shaft, the base of the penis must be adhered to the pubic symphysis so as to avoid future retraction of the penis and recurrent entrapment.^3^^,^^8^ This can be accomplished in multiple ways. Figler et al^3^ reported exposing the periosteum overlying the pubic symphysis to ensure the skin adjacent to the base of penis is directly attached to the periosteum. In this case, 5 interrupted no. 1 Prolene sutures placed first into the periosteum and then into the dermis of the skin bridge cephalad to the base of the penis. Following this, 2 closed suction drains were placed in the panniculectomy bed and the skin was closed in 2 layers. Rybak et al^8^ reported fixing the base of the penis to 4 points of the pubis, specifically at the 2, 4, 8, and 10 o'clock positions. The base of the penis was fixed with 2-0 Ethibond sutures. Both procedures result in adequate outcomes, and no recurrent entrapment of the penis was noted.

**Figure 8:**
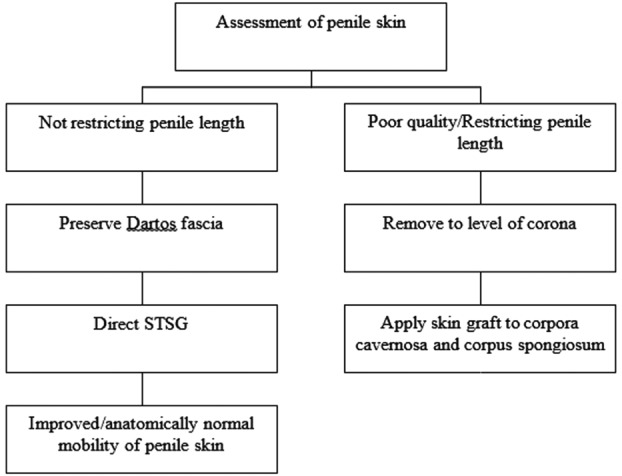
Algorithm determining viability and management of penile skin following fixation of the adjacent skin to the pubis. STSG indicates split-thickness skin graft.

Once the skin adjacent to the penis is fixed appropriately to the pubis, an important distinction must be made regarding the viability of the penile skin (Fig 8). If the skin is poor, yet Dartos fascia is viable, Dartos fascia should be preserved and the split-thickness skin graft is adhered directly to Dartos fascia. This allows for improved mobility of the grafted skin over the penile shaft and results in the most anatomically correct penis. If penile skin is very poor or restricting length, penile skin should be removed to the level of the corona to avoid distal lymphedema. Placing the grafted skin directly to the corpus cavernosum and corpus spongiosum is acceptable; however, this results in a less anatomically correct penis.

Whether removing the penile skin to the level of the corona or Dartos fascia, the size of the skin graft must be determined. The length of the erect penis must be known to determine the exact size of the skin graft needed; however, the most accurate and efficient way of determining this is unknown. It has been reported that the stretched penile length strongly correlates with erectile length and that this method is sufficient to determine the size of the skin graft needed.^3^ It is also possible to artificially erect the penis with prostaglandin E_1_ intraoperatively and thus adhere the skin graft directly to the erect penis.^11^ With postoperative tadalafil application and negative pressure dressings, patient outcomes have been reported to be successful in that patients had stable skin grafts 2 weeks postoperatively and were able to have sexual intercourse at 2 to 3 months.^11^

In using a split-thickness skin grafts, there are various approaches depending on surgeon preference, but it is preferred to use split-thickness skin grafts over trying to preserve native penile skin. Trying to preserve native penile skin can result in infection and restricted penile length.^1^^,^^3^ Grafts were taken from the anterior or lateral thigh with a dermatome, measuring 0.014 to 0.018 in deep and can be either meshed or unmeshed.^3^ In 2 reports, a thin layer of fibrin glue was sprayed under the graft, stating that this enhances graft take and decreases grafted skin contracture.^12^^,^^13^ The graft can be secured to the penis with 4-0 chromic sutures with the suture line on the ventral aspect of the penis to mimic the ventral raphe.^3^^,^^8^ Suture tails can be left long to tie over the bolster dressing, or the bolster dressing can be secured to the penis with interrupted 2-0 silk sutures at the proximal and distal aspects of the graft.^3^^,^^8^

Patients’ hospital stay has been reported to be as little as 2.25 days or as long as 6.6 days and while management is complicated, patients are able to regain sexual and urinary functions.^1^^,^^3^ There has been limited reports of patients who are unsatisfied postoperatively. Patients should receive low-molecular-weight heparin, if possible, throughout their hospital stay to prevent the formation of deep vein thrombosis.^3^ Major long-term complications besides those described earlier have not been reported, but it must be noted that the most prevalent are wound dehiscence and infection.^1^^,^^3^^,^^8^ Depending on the severity of the infection, operative debridement may be necessary; however, conservative management with antibiotics has been sufficient if the infection is not severe.^8^ With negative pressure dressing placed into the wound bed, patients have more open drainage and reduced chance of abscess formation. Specifically, open wound management is thought to decrease recovery time by decreasing the chances of wound dehiscence seen with a primary closed wound. There has been no statistical significance recorded of which is preferable regarding open wound management versus a primary closed wound.^9^ These results are a product of small study population and thus a larger study population is needed to solidify the significance of which closure is preferable in reducing postoperative complications.

## SUMMARY

Management of a buried, hidden penis is complex and difficult. Patients are often, but not always, obese and have poor hygiene due to the inability to cleanse areas that are entrapped by excessive fat. These patients are unable to stand to micturate, as urine dribbles out of dimpled areas, and are unable to engage in sexual intercourse. Patients are often depressed; however, depression almost entirely improves postoperatively.^1^^,^^8^

Following removal of the overhanging panniculus or massive scrotal lymphedema, satisfactory reconstruction of a hidden penis is possible when proper care is taken to adhere the base of the penis to the pubis. Split-thickness skin grafts are often necessary but depend on the viability of the penile skin and whether it is restricting penile length.

Complications with wound dehiscence and infection are not uncommon but can be managed with antibiotics or with debridement and local wound care in severe cases. Patients generally recover well, are satisfied with results, and are reported to have fully regained urinary and sexual functions following surgical correction of the buried penis.^1^^,^^3^
